# Supplementary material for: Patterns of Intron Gain and Loss in Fungi
Source: PLoS Biol. 2004 Nov 30;2(12):e422. doi: 10.1371/journal.pbio.0020422 (PMC532390; doi:10.1371/journal.pbio.0020422)
Supplement: Table S1 — Also available at http://genes.mit.edu/NielsenEtAl/. (4.3 MB ZIP). [file pbio.0020422.st001.zip › NielsenEtAl/html/1422.html]

AN5722.1.NCU01767.1.MG03154.1.FG05205.1


```
 CLUSTAL W (1.82) Multiple Sequence Alignments - Introns Inserted


Sequence 1: NCU01767.1	622 aa
Sequence 2: FG05205.1	594 aa
Sequence 3: MG03154.1	620 aa
Sequence 4: AN5722.1	596 aa
Alignment Length: 648 aa
Number Identitical Residues: 266 aa
Alignment Score (without introns) 12777


MG03154.1 	MRSRNAKALCTATYQLAVRSITRNTARTKPGFSLVGSCSTPAARKSIALRRLYSSHSRTR
NCU01767.1	MRRAAVKALR-ASYQCTCRGLRVSSRGSPLPLLQSSKAASSGSPTAHYNPRLGTATARYL
FG05205.1 	MNRVAVKSLR-TTSLSTVHALPRAPRRG---------FSISSTVLAHQSRSKASAHARYA
AN5722.1  	MRRAALQALRSSRRAPAWRVGGRTPPIS-------FTSSPSLGLRSYSTPKLSAS-----
          	*.    ::* ::    : :     .           . : .    :       ::     

MG03154.1 	TTR--------PEHAPVNQQLLTSSMSVRRFSLALVSGLVG-YGAWYSYRDSSTDATAAT
NCU01767.1	SSTGARDSSGTHHHSNARPEPLRSSMYVRGAFVTLLSGLVA-YGAWY----NSGDSNSES
FG05205.1 	------------HRKSTKHIPLKSSMYFRRLPTALLSSIVVGYGAWYSY--NSTDSVPTS
AN5722.1  	---------------------LQSSMHLRTFSVAAVSAVVA-SGAWYAYQGDGSQTPAAA
          	                     * *** .*    : :*.:*   ****:  ... :: . :

MG03154.1 	RAFTNSASNPTKAAVNG-AAPTRNVLVITADELYTSTFVGEGPISKITDDERRMVVEMLN
NCU01767.1	---PIASTTSATTTATG-AVPTRSVLVIGADELHTGTFVGDGPISKTTSDN-ERVVEMLS
FG05205.1 	---TFSANSLTSTQTAD-AVPTRTVLVVGADELRQGTIVGEGPISKATSDDGRRIVEMLT
AN5722.1  	GTSSQTRSFTSTAHAEQPSEPSRRALLVDNDQFYTATLSGEQPLQKNTDDSDRRLLEMLT
          	 : . : .  :.: .  .: *:* .*::  *::  .*: *: *:.* *.*. . ::***.

MG03154.1 	PEQSTQKLRKNEQSYIVNRGQGVVRYDLVQLPSNDPIEDDHAEKIVEVPDGSQPSSKN--
NCU01767.1	PEQATRKLRQYEQSVYVNRGQGVVRYDVAQLPSNDPIEDDHAEKIVEVPN-KAPSSDPS-
FG05205.1 	PDQATQKLRRLEQSYSVSRGQGVTRYDLVQLPSNDPIEDDHAEKIVQVPSRSSAETDNS-
AN5722.1  	PEQATQKLRKNEESYLVNRGKGVVRYDVVQVPSNSPIEDDHAEKIVEVPASTSAANEGQS
          	*:*:*:***: *:*  *.**:**.***:.*:***.***********:**  . . .. .:

MG03154.1 	--DWMFWGVFDGHS2GWTTSAKLRQALIGSVARELNDTYRAAPS----MTPTPEAIEAAM
NCU01767.1	--DWMFWGVFDGHS2GWTTSAKLRQALISFVARELNETYKSSPD----LIPSAAAVESAI
FG05205.1 	--DWMFWGVFDGHS2GWTTSATLRESLINYVARELNATYKLASG----ELPSEDAVTQAI
AN5722.1  	SSDWMFWAVFDGHS2GWTTSAKLRNVLISYVARELNATYKSASSDPSLVLPSSEAVDAAI
          	::*****.****** ******.**: **. ****** **: :....:   *:  *:  *:

MG03154.1 	KTGFVRLDDEIVNQSVEKVLKQNNKTVAAELLAPALSGSCALLSFYDSRTGLLRVACTGD
NCU01767.1	KTGFTRLDDEIVHQSVQRVLKSNNRLVAAEHLAPALSGSCALLSFYDSKSKLLRVACTGD
FG05205.1 	KTGFNNLDNEIVHKSVEKVFKGGSKTVAAELLQPALSGSCALLSFYDSRSNLLRVACTGD
AN5722.1  	KQGFVRLDNDIVHGSVNQVFKSNSRRAAAELLAPALSGSCALLAFYDSQTRDLKVACAGD
          	* ** .**::**: **::*:* ..: .*** * **********:****::  *:***:**

MG03154.1 	SRAVLGRRSASSDKWTATPLSTDQTGANPEEAARMRKQHPGEEHVVRNGRVLGGLEPTRA
NCU01767.1	SRAVLGRRSESG-KWTATALSTDQTGSNPDEAARLRKLHPGEEHVVRHGRVLGGLEPTRA
FG05205.1 	SRAVLGRRAANG-KWTATALSDDQTGSNPSEVDRMRKEHPGEENVIRNGRVLGGLEPSRA
AN5722.1  	SRAVLGRRSENG-KWTATPLSEDQTGGTPSEMKRLREEHPGEPNVVRNGRILGQLEPSRS
          	********: .. *****.** ****..*.*  *:*: **** :*:*:**:** ***:*:

MG03154.1 	FGDASYKWTRDVSERLRRSFFGRTPSALLRTPPYVTAEPVVTTTKIEPQNGDFLVLATDG
NCU01767.1	FGDASYKWSRELSEKLREKFFGRSVSPLLKTPPYVTAEPVVTTTKIEPEKGDFVVMATDG
FG05205.1 	FGDAVYKWSRDVAYKLRENFFGRSPSPLLKTPPYVTAEPVVTTTKVSPENGDFLVLATDG
AN5722.1  	FGDAFYKWSKETQEKIKRQFFGRTPHPLLKTPPYVTAEPIITTTKVDPSQGDFLVLATDG
          	**** ***:::   :::..****:  .**:*********::****:.*.:***:*:****

MG03154.1 	LWEMLTNEEVVGLVGKWLETQ-QAGGSGS---SYWSKMFGGSSTKSGLPVEAVGSKDSAG
NCU01767.1	FWEMLTNEEVVGLVGKWIES---QAGSSSKSSGYFSFLQKGS--KTALPVESSESEKNSG
FG05205.1 	LWEMLTNEEVVGLVGKWIETE-GQSGTNSQFDAAWDRIFGSS--KTPLPVEESKTAGPDG
AN5722.1  	LWEMLSNEEVVGLVGQWIEEQKAAAGAGTGNKSWVRSLFGSQ--PTQLPVEAPKETSTDG
          	:****:*********:*:* ..  .*:.:  ..    :  ..   : ****        *

MG03154.1 	GQKTPIRVQQWGLS-PDGKDRFVVQDKNVATHLVRNALGGKNEEQVSALLTLPSPFSRRY
NCU01767.1	-NKTPIRQRQWGATGTDPMERFVVQDKNVATHLVRNALGGKNQEQVSALLTLPSPFSRRY
FG05205.1 	-NKTPIRVQQWGID-PDAKDRFTVKDKNVATHLIRNALGGNNDEQVCALLTLPSPFSRRY
AN5722.1  	-QRRPIRQQQYDIS--GAASRFVVEDKNAATHLVRNAMGGKDKDMLCALLTLPSPYSRRY
          	 :: *** :*:.    .  .**.*:***.****:***:**::.: :.********:****

MG03154.1 	R2DDLTVQVIFFGNEGEKTGDVVVNEEATSMP-----GSSSQAAAAKAKL
NCU01767.1	R~DDLTVQVIFFGYDGPKTGEIVVNKEATAAALAAAAADGNKTGPVKPKL
FG05205.1 	R2DDLTVQVIFFGH-GEKTGEITVNLEATAG-------EG-----VKAKL
AN5722.1  	R2DDVTVEVIFFGE-SPDNRTISINEEASASE-----------DNIKAKL
          	* **:**:*****  . ..  : :* **::                *.**
```
